# Multi-Step Extracellular Matrix Remodelling and Stiffening in the Development of Idiopathic Pulmonary Fibrosis

**DOI:** 10.3390/ijms24021708

**Published:** 2023-01-15

**Authors:** Constança Júnior, Anna Ulldemolins, Maria Narciso, Isaac Almendros, Ramon Farré, Daniel Navajas, Javier López, Mar Eroles, Felix Rico, Núria Gavara

**Affiliations:** 1 Unitat de Biofísica i Bioenginyeria, Facultat de Medicina i Ciències de la Salut, Universitat de Barcelona, 08036 Barcelona, Spain; 2 The Institute for Bioengineering of Catalonia (IBEC), The Barcelona Institute of Science and Technology (BIST), 08028 Barcelona, Spain; 3 CIBER de Enfermedades Respiratorias, 28029 Madrid, Spain; 4 Institut d’Investigacions Biomèdiques August Pi i Sunyer, 08036 Barcelona, Spain; 5 Institut Pasteur de Lille, U1019-UMR9017-CIIL-Centre d’Infection et d’Immunité de Lille, Université de Lille, CNRS, Inserm, CHU Lille, 59000 Lille, France; 6 Aix-Marseille, CNRS, INSERM, LAI, Centuri Centre for Living Systems, 13009 Marseille, France

**Keywords:** extracellular matrix, atomic force microscopy, mechanosensing, idiopathic pulmonary fibrosis

## Abstract

The extracellular matrix (ECM) of the lung is a filamentous network composed mainly of collagens, elastin, and proteoglycans that provides structural and physical support to its populating cells. Proliferation, migration and overall behaviour of those cells is greatly determined by micromechanical queues provided by the ECM. Lung fibrosis displays an aberrant increased deposition of ECM which likely changes filament organization and stiffens the ECM, thus upregulating the profibrotic profile of pulmonary cells. We have previously used AFM to assess changes in the Young’s Modulus (E) of the ECM in the lung. Here, we perform further ECM topographical, mechanical and viscoelastic analysis at the micro- and nano-scale throughout fibrosis development. Furthermore, we provide nanoscale correlations between topographical and elastic properties of the ECM fibres. Firstly, we identify a softening of the ECM after rats are instilled with media associated with recovery of mechanical homeostasis, which is hindered in bleomycin-instilled lungs. Moreover, we find opposite correlations between fibre stiffness and roughness in PBS- vs bleomycin-treated lung. Our findings suggest that changes in ECM nanoscale organization take place at different stages of fibrosis, with the potential to help identify pharmacological targets to hinder its progression.

## 1. Introduction

Idiopathic pulmonary fibrosis (IPF), the most common and severe form of pulmonary fibrosis, is a chronic and progressive disease affecting the lungs, which commonly results in fatal outcomes. It is characterised by irreversible loss of pulmonary function due to destruction of tissue architecture, and its causes remain unknown. If left untreated, IPF has a median life expectancy of 2–3 years after diagnosis. Despite recent advances in anti-fibrotic drug development, the current available therapies are only capable of slowing down the progression of the disease. The only reliable treatment for IPF is still lung transplantation [1,2,3,4].

The formation of temporary fibrotic tissue in the lung is a common phase of the tissue’s repair response to wound healing. In injured tissues, fibroblasts are activated and differentiate into myofibroblasts, which then deposit extracellular matrix (ECM) components, such as collagen and elastin. When the damage is minor and non-repetitive, wound healing ceases once the process resolves, resulting only in a temporary increase in the accumulation of ECM components and subsequent apoptosis of myofibroblasts. In the case of IPF, the wound healing pathway is compromised, resulting in a runaway engagement of the profibrotic feedback loop, where the formation of fibrotic sites triggers a continuous recruitment and accumulation of activated fibroblasts. These events result in a continuous remodelling and compromise the ECM, which can ultimately lead to organ failure [5,6,7,8,9].

Mechanotransduction has a pivotal role in the onset and progression of several respiratory diseases. Changes in the ECM result in aberrant mechanical signaling that alters cell proliferation, migration, differentiation and activation [10,11,12]. High mechanical stress and the presence of specialised matrix proteins, such as fibronectin, are known to be main factors that drive the differentiation of fibroblasts to myofibroblasts [13,14]. Thus, in order to fully understand the pathophysiology of IPF and possible targets for the development of effective therapies, it is of the utmost importance to understand the changes undergone by the ECM at the relevant cell-sensing scale and from a physical point of view [15,16,17,18,19].

The mechanical response of the ECM is mainly determined by the relative disposition and network structure of its constituents. A common approach to assess these micromechanical features is the use of atomic force microscopy (AFM). AFM is a technique that allows high-spatial resolution, nanomechanical mapping of heterogeneous interfaces. Samples are probed with a small tip, and by recording the force applied and the resulting deformation of the surface of the sample under the probe, it is possible to obtain a force-indentation curve. The resulting force-indentation curve is then fitted to an appropriate contact mechanics model and the apparent Young’s Modulus of the sample is estimated [20,21,22,23]. By monitoring also the height at which the tip first becomes in contact with the sample, it is possible to obtain simultaneous topography and mechanical maps of the sample (known as force or mechanical mapping or quantitative imaging (QI) mode). This allows to directly correlate mechanical and morphological features of the ECM. On the other hand, AFM protocols can also be designed to determine the viscoelastic response of the sample, by applying low-amplitude oscillatory forces at different frequencies and estimating the frequency-dependent complex shear modulus ($G^{*}$) [24]. Viscoelasticity is an intrinsic feature observed in many biological tissues, and it is thought to arise from the major polymeric tissue components and their interactions, namely collagen, elastin, and proteoglycans. Thus, the use of AFM to probe the topography and viscoelasticity of the ECM at the nanoscale provides essential information on the remodeling that the ECM goes through throughout the progression of IPF.

One of the main challenges when studying the morphological and mechanical properties of the ECM in IPF is the high spatial heterogeneous nature of the disease [25]. Given the need to acquire dense datasets, efficient and robust tools to process and analyse them must be developed alongside. Here, we propose a novel data analysis approach to AFM data based on a multidimensional exploratory analysis of different features, by running a rolling window through mechanical maps and applying Fourier Transform and Machine Learning methods. By coupling these techniques with conventional ones, we have herein been able to perform for the first time an extensive topographical and viscoelastic analysis at the micro- and nano-scale of the ECM throughout different stages of development in IPF. To that end, we have used a bleomycin-induced murine model of IPF. Interestingly, our findings suggest different stages on the re-arrangement of the ECM components, linked to the different steps of IPF development. In particular, we identify a switch regarding the correlation between surface stiffness and roughness, which is positive for the PBS-instilled lungs and negative for the bleomycin-instilled lungs. As such, we propose that these ECM stages might serve as key targets to stop the progression of the disease.

## 2. Results

### 2.1. Fibrosis Leads to ECM Network Stiffening at Late Stages of Development

**Figure 1:**
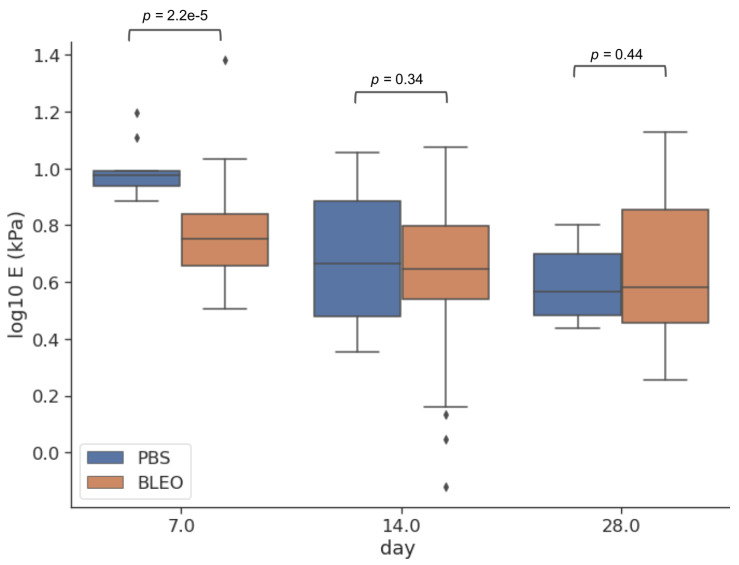
Young’s Modulus ELR measured by AFM. Boxplots represent the distribution of the data at days 7, 14, and 28 after instillation. The centre line in the boxplots represents the median value, the top and bottom limits of the box represent Q1 and Q3 of the data. Whiskers represent minimum and maximum values, the diamonds represent outliers and are data points outside 1.5 times the interquartile range. (*n* = 15 for control and *n* = 35 for fibrotic, corresponding to 5 maps per animal.)

After decellularizing the ECM slices, and before carrying out any measurements, phase contrast imaging was used to confirm that the decellularization protocol preserved the internal micro-architecture of the lung. Initially, we carried out exploratory low-resolution AFM elasticity maps using a probe with a large spherical tip (radius = 5.5 μm) to determine the overall stiffness of the ECM. The large contact area allows for a combined assessment of the mechanical response of the various fibres composing the ECM network and their interaction. The Young’s modulus *E* was used as a measure of the stiffness of the ECM (Figure 1). From now on, this will be designated ELR (*E* at low resolution). We found that the median values for ELR for the control samples are 9.5 kPa (Q1–Q3 being 8.5–9.8 kPa), 4.5 kPa (Q1–Q3 being 3.0–7.8 kPa) and 3.6 kPa (Q1–Q3 being 3.0–5.0 kPa) for days 7, 14, and 28 after PBS instillation, respectively. After day 7, there is a softening of the lung ECM over time, likely due to the resolution of an initial inflammation caused by the insertion of liquid into the animal’s lungs. In contrast, we found that the median values for *E* for the fibrotic samples were 5.6 kPa (Q1–Q3 being 4.6–6.9 kPa), 4.5 kPa (Q1–Q3 being 3.5–6.3 kPa), and 3.8 kPa (Q1–Q3 being 2.9–7.1 kPa) for days 7, 14, and 28 after bleomycin instillation, respectively. The same process of softening after bleomycin being instilled into the lungs can be observed, with the control lungs being initially slightly stiffer until a turnover is seen at day 28 at late stage of fibrosis. Together, these results suggest that there are two distinct mechanical responses: the first one corresponding to an initial response to the insertion of a foreign liquid into the lungs, that becomes resolved in the PBS instilled lungs, and the second one, which becomes more prominent in later stages of fibrosis development.

### 2.2. Local Fibre Stiffening Precedes Gross Network Stiffening in Fibrotic ECM with No Changes in Network Surface Topography

**Figure 2:**
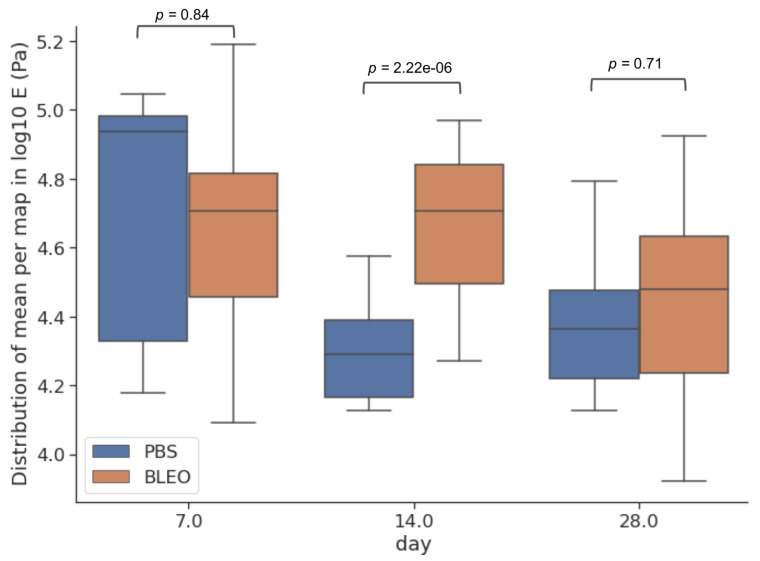
Young’s modulus EHR assessed by AFM through high-resolution maps. The centre line in the boxplots represents the median value, the top and bottom limits of the box represent Q1 and Q3 of the data. Whiskers represent minimum and maximum value of the data, the diamonds represent the outliers and are data points outside 1.5 times the interquartile range. (*n* = 9 for control and *n* = 21 for fibrotic, corresponding to 3 maps per animal).

**Figure 3:**
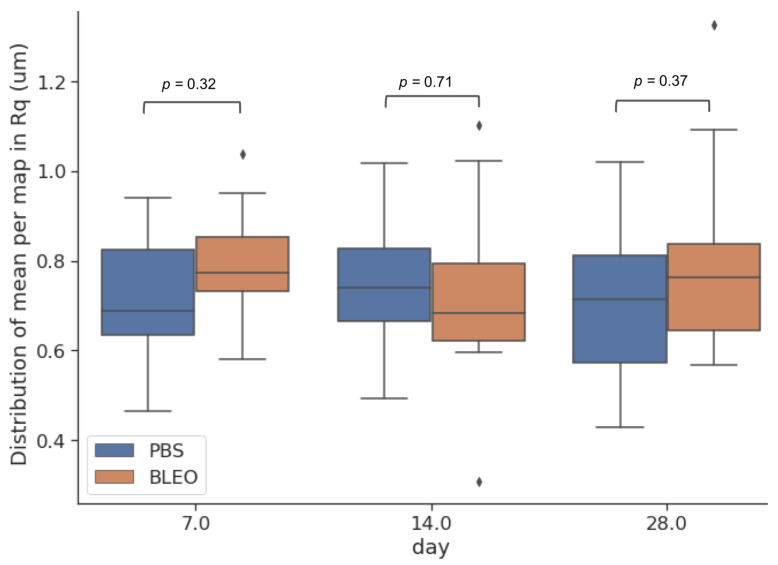
Surface roughness $R_{q}$ calculated from local height variations of the high-resolution maps. The centre line in the boxplots represents the median value, the top and bottom limits of the box represent Q1 and Q3 of the data. Whiskers represent minimum and maximum value of the data, the diamonds represent the outliers and are data points outside 1.5 times the interquartile range. (*n* = 9 for control and *n* = 21 for fibrotic, corresponding to 3 maps per animal).

**Figure 4:**
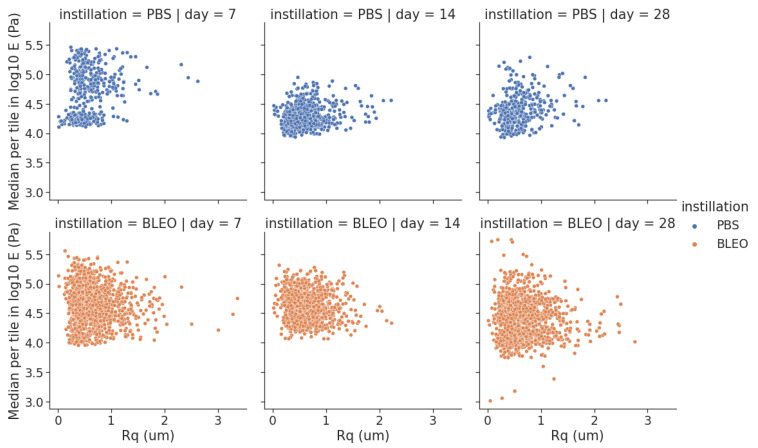
Relationship between distribution of the median EHR and $R_{q}$.

**Figure 5:**
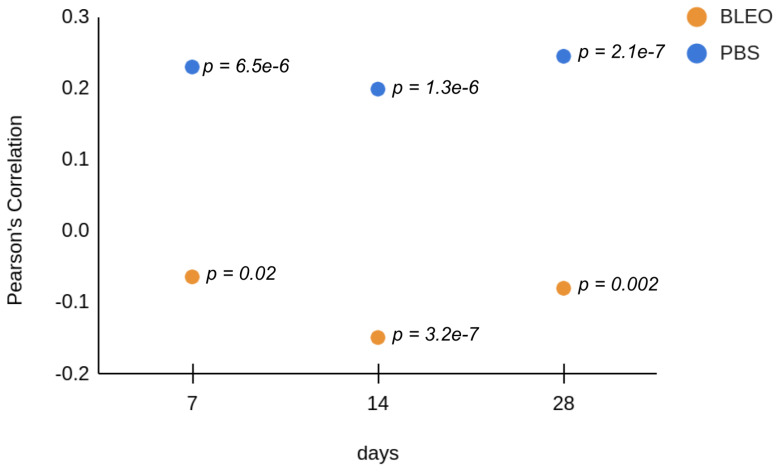
Pearson’s correlation between median values of EHR and $R_{q}$.

**Figure 6:**
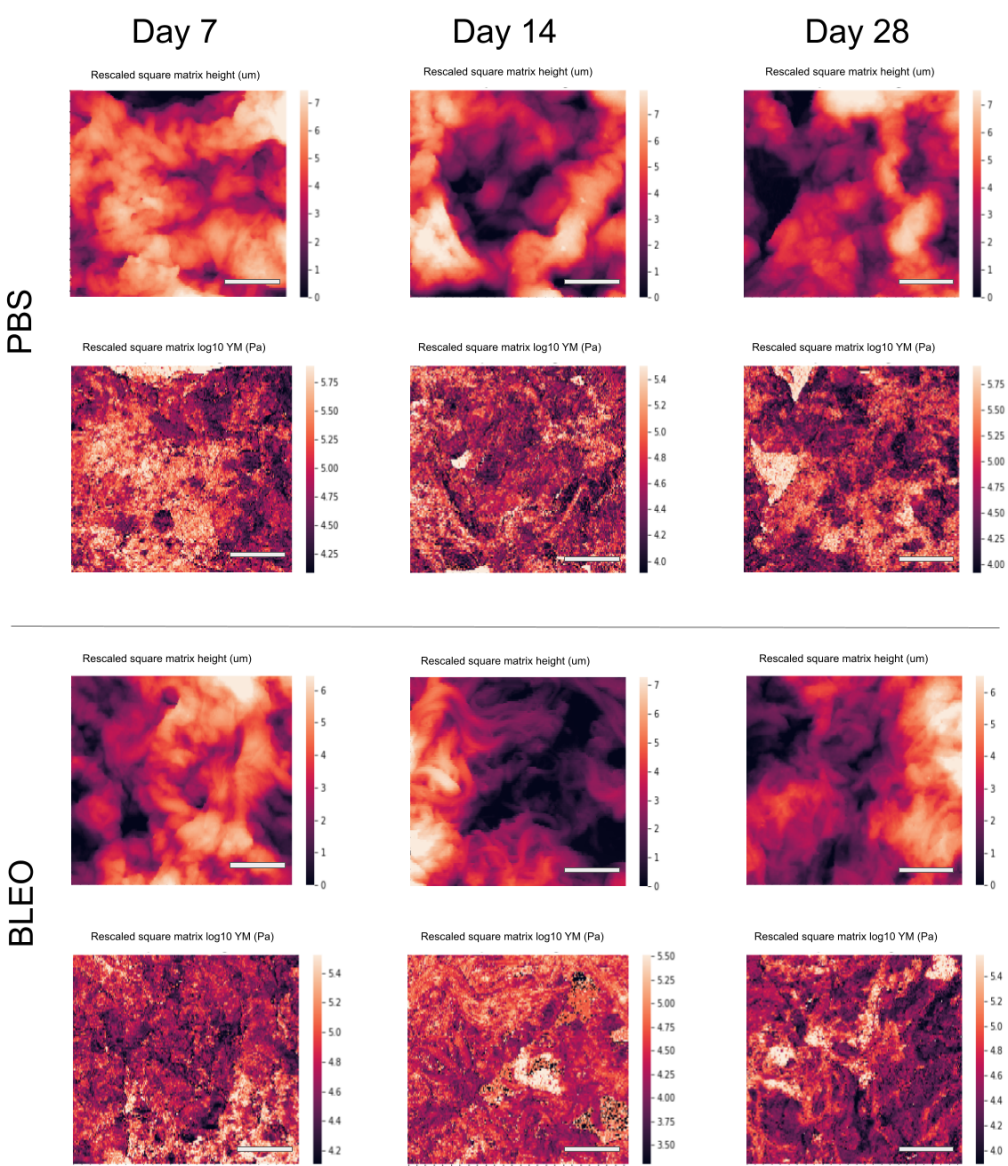
QI images of decellularized ECM from PBS-instilled (**top panel**) and bleomycin-instilled lungs (**bottom panel**). Top rows of each panel correspond to the height profile and bottom rows correspond to elasticity profile. Scale bar = 10 μm.

To investigate the changes to which the fibres constituting the network go through and further explain the alterations on the mechanical response of the ECM due to fibrosis, high-resolution QI maps were performed. We analysed the changes in stiffness by taking one median value of *E* per tile in each QI elasticity map, to which we will refer as EHR from now on (Figure 2). The distribution of the medians showed for the controls a median value of EHR of 83.17 kPa (Q1–Q3 being 28.2–93.3 kPa), 19.5 kPa (Q1–Q3 being 16.6–27.0 kPa), and 25.1 kPa (Q1–Q3 being 16.2–32.4 kPa) for days 7, 14, and 28 after PBS instillation, respectively. These results follow the same tendency of softening with time observed in the results obtained from the low resolution maps. On the other hand, the values of EHR obtained for the lungs treated with bleomycin were 52.5 kPa (Q1–Q3 being 30.2–67.6 kPa), 51.3 kPa (Q1–Q3 being 33.1–69.2 kPa), and 30.9 kPa (Q1–Q3 being 17.8–44.7 kPa) for days 7, 14, and 28, respectively. These results show that at the nanoscale, the stiffening of the fibres compared to control is kept after day 7 and remains stiffer through time. Analogously, we performed the analysis of the distribution of the surface roughness $R_{q}$ per tile in each QI height image (Figure 3). We obtained a median value of $R_{q}$ for the control samples of 0.69 μm (Q1–Q3 being 0.64–0.82 μm), 0.74 μm (Q1–Q3 being 0.67–0.83 μm), and 0.71 μm (Q1–Q3 being 0.57–0.81 μm) for days 7, 14, and 28 after PBS instillation, respectively. Regarding the fibrotic samples, we obtained for $R_{q}$ the median values of 0.77 μm (Q1–Q3 being 0.73–0.85 μm), 0.68 μm (Q1–Q3 being 0.62–0.79 μm) and 0.76 μm (Q1–Q3 being 0.64–0.83 μm) for days 7, 14, and 28, respectively. Interestingly, when looking into the Pearson’s correlation between surface roughness $R_{q}$ and and the stiffness EHR at each tile (Figure 4 and Figure 5), we found that for the PBS-treated lungs, the correlation between surface roughness and fibre stiffness is positive while, for the bleomycin-treated lungs, this correlation is negative. In fact, observing the QI height images (Figure 6), at day 14 and 28, there seems to be a thinning of the fibres for the bleomycin-treated lungs, whilst no noticeable differences are observed in the control samples with time. If the thickness of the fibres decreases, the apparent surface roughness will increase. A possible interpretation for the positively correlated values is that, if the fibres are thicker, the apparent roughness in the tile will be lower, thus the stiffness being measured will belong to less number of fibres. An increase in roughness could mean that in that tile, more fibres and/or entanglements are present, thus increasing the roughness with stiffness. On the other hand, for an ECM with looser and thinner fibres, an increase in roughness can indicate thinner and/or less entangled fibres, which considering the tile analysis, would correspond to an area with lower stiffness.

Regarding the PCA analysis of the images, the algorithm found more components present in the images corresponding to the bleomycin-treated lungs than the control ones. We found a mean number of 3 ± 0.3, 2 ± 0.1, 2 ± 0.2 components for the control samples at days 7, 14, and 28, respectively. For the fibrotic samples, we found that the mean number of components identified were 3 ± 0.3, 3 ± 0.2, and 3 ± 0.2 for days 7, 14, and 28, respectively. The components can be interpreted as an index of the organisation and composition of the network. This means that, for each component identified, a structure that introduces a significant amount of variability to the image is associated with said component. These results could indicate a change in either the thickness, fractality, or directionality of the fibres in the fibrotic lungs, which can be translated into different mechanical responses dependent on network organisation and are in accordance with the results for the opposite correlation observed between stiffness and surface roughness.

### 2.3. Fibrosis Progress Follows a Fluidization of the Network

**Figure 7:**
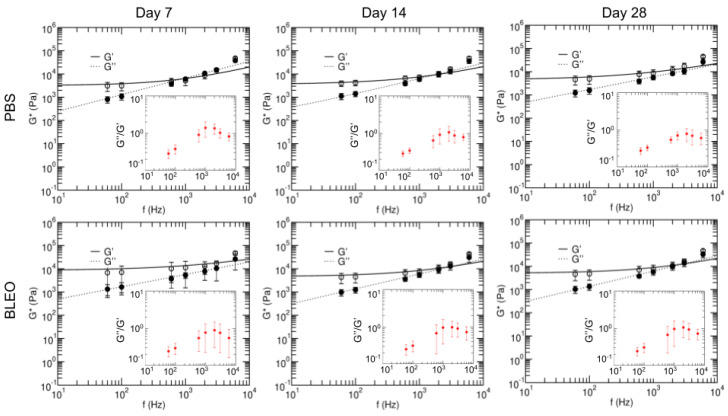
Frequency-dependent complex shear modulus and loss tangents of the lungs at 7 (**left panel**), 14 (**center panel**) and 28 (**right panel**) days after instillation with PBS (**top row**) and bleomycin (**bottom row**). Open symbols represent G′ and closed symbols, G″. Solid and dashed lines are fits of the two-power-law model. Data are mean ± SD. Fit parameters are shown in Table 1.

To further characterise the mechanical response of the ECM at the microscale, we next focused on understanding its viscoelastic behaviour. We used AFM to measure the complex shear modulus of the lung slides at different probing frequencies (Figure 7).

$G^{*}\left(f\right)$ was modelled using a double power-law (Equation (5)), where a weak power law is observed at low frequencies, followed by a stronger power law at high frequencies. The coefficients A and B can be interpreted as an index of stiffness associated to the power law exponents (α and β) at low and high frequencies, respectively. Coefficient A is about two orders of magnitude larger than B and higher for the fibrotic than the control samples at all time points. Coefficient B increases with time for the control samples, whilst the opposite trend is observed for the fibrotic samples (Table 1). This indicates that the bleomycin-treated samples are apparently stiffer at low frequencies. At high frequencies, PBS-treated samples apparently stiffen with time, while bleomycin-treated samples soften with time. This, followed by the observation that the exponent beta follows an opposite trend regarding its change with time for the PBS-treated lungs in comparison to the bleomycin lungs, suggests that changes on the conformation of the network might be already compromised in early stages of fibrosis. This change could be associated to mechanical signalling involved in the resolution of an inflammation-like response due to the intratracheal instillation.

Based on the two-power-law model, we further defined the transition frequency ($f_{t}$) at which the material goes from being dominated by a more solid-like regime to a more fluid-like regime. We found that $f_{t}$ increases at later time points in regards of day 7 for the PBS-treated lungs, but the opposite trend is observed for the bleomycin-treated lungs. These findings suggest that a step towards the resolution of the initial inflammatory phase involves the solidification of the ECM, but in the case of fibrosis, the matrix fails to restore its mechanical homeostasis.

### 2.4. Decrease in Fibronectin Content Is Simultaneous with Softening of the ECM

**Figure 8:**
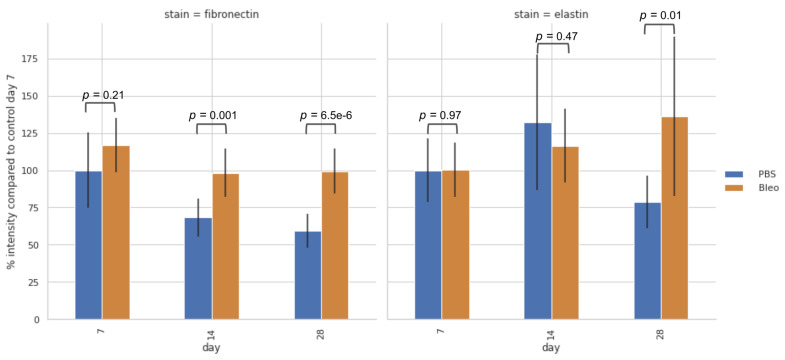
Evolution of percentage of fluorescence signal in relation to PBS instilled samples at day 7 for fibronectin (**left**) and elastin (**right**). Data are mean ± SD (*n* = 3 for control and *n* = 7 for fibrotic).

**Figure 9:**
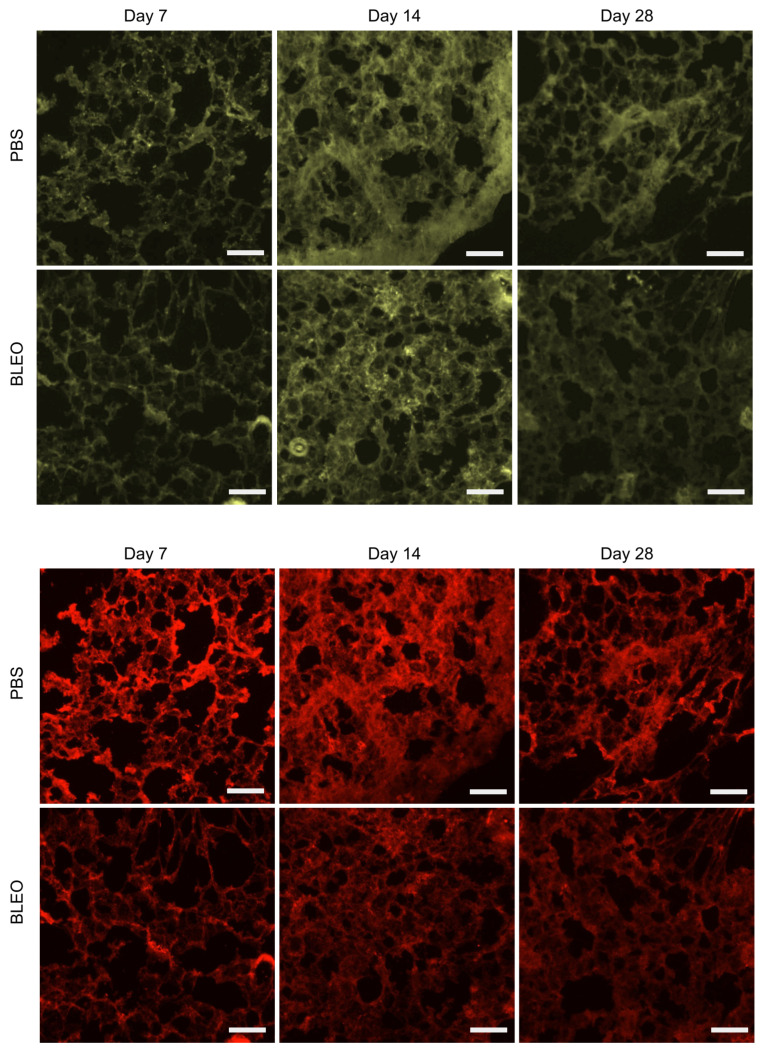
Epifluorescence images of immunostainings of elastin (**top**) and fibronectin (**bottom**) performed on PBS-instilled and bleomycin-instilled decellularized lung ECM. Scale bar = 50 μm.

Fluorescence images of immunohistochemically-stained lung slices were collected and quantified to assess whether there had been alterations in the relative quantity of ECM proteins (Figure 8 and Figure 9). The amount of fibronectin is higher for the bleomycin-treated lungs in regard to the PBS-treated lungs for all timepoints. For the PBS-treated lungs, the amount of fibronectin decreases with time, whilst for the bleomycin-treated lungs it decreases at day 14 and increases slightly at day 28. Interestingly, these results are analogous to the ones observed for the ELR maps. These results suggest that fibronectin assumes a determinant role in regard to the changes in the mechanical response of the ECM at the network level.

Regarding the relative amounts of elastin, no differences between PBS- or bleomycin-treated samples could be observed at day 7. They later increase at day 14 for both PBS- and bleomycin-treated lungs, being higher for the control samples than the fibrotic ones. Finally, the relative amount of elastin lowers for the PBS-treated samples at day 28 achieving its lower value, but achieves its higher value for the bleomycin-treated samples at the last time point. As elastin is the main component in the lung responsible for its elastic recoil, changes in its relative amount highly influences the mechanical response of the lung at both the fibre and network level. The changes observed could indicate that elastin might have an important role in modulating the mechanical homeostasis of the lung, and, consequently, on determining the development and resolution of fibrosis.

Looking into Pearson’s correlation between the mean values for ELR maps and the absolute value for the signal obtained from the fluorescence images, we obtain a value of −0.093 for elastin and 0.437 for fibronectin. These results indicate that the presence of fibronectin influences the stiffness of the ECM, which is an indication that it poses as an important component in determining the elastic properties of the network.

## 3. Discussion

In this work, we carry out an extensive micromechanical characterization of the decellularized lung ECM in IPF through time, unravelling previously unknown alterations in the progress of the mechanical changes of the fibrotic ECM. At the microscale, we find that the ECM softens after an initial instillation-induced response phase but at later time points fibrosis becomes predominant and maintains a stiffer profile than its healthy counterpart, suggesting a switch in the resolution of an initial inflammatory-like response that determines further development of fibrosis. Further examination of the mechanical alterations of the fibres constituting the ECM showed that in bleomcyin-instilled lungs, the stiffening of the fibres at the inflammatory-like phase is kept from early stages through late stages of fibrosis, whilst the fibres belonging to healthy lungs are able to resolve their initial inflammatory state. These results suggest that an alteration at the conformation of the fibres takes place due to inflammation, which, if not resolved, results in the development of fibrosis. We then characterised the morphological profile of the ECM, which showed that fibres belonging to ECM from bleomycin-treated lungs become thinner and do not entangle as much as the control networks. Moreover, immunohistochemical staining showed a positive correlation between relative fibronectin content with the stiffness assessed through LR elasticity maps. Further micro-rheology experiments showed that the ECM solidifies upon resolution of the inflammatory-like phase, but fails to do so in the bleomycin-treated ECM. These results together point towards a key change on the organisation of the ECM components due to fibrosis, beyond the simple increase in filamentous matrix deposition.

Bleomycin induces temporary lung fibrosis in mice and rats, and has been widely used in several studies as a model to study mechanisms related to IPF and PF [26,27,28,29]. Typically, the control population in these studies are instilled with a vehicle saline solution. Although no inflammation markers or significant changes in histology have been seen in other studies after instillation of vehicle solution, these are usually assessed after there would have been enough time for fibrosis to develop. As instillation of a liquid in the lungs is an invasive procedure, it remains unclear whether there is an early inflammatory response shortly after the instillation that could affect the tissue. In fact, a study by Numano and colleagues [30] comparing the effects of instilling intratracheally different vehicles in rats showed that for all vehicles, including PBS, there is an increase in the presence of alveolar macrophages in a histopathological examination of the lungs. Inflammation induces tissue stiffening, which, in healthy conditions, ends up being resolved as the inflammation subsides. However, when the acute inflammatory phase fails to be resolved, it develops into chronic inflammation. Fibrosis is a product of the permanent tissue remodelling associated with unresolved inflammation [31]. In fact, several studies have shown that inflammation and the presence of inflammatory-related proteins can increase tissue stiffness preceding the development of fibrosis [32,33,34]. Our results further confirm these observations, and highlight the pro-inflammatory conditions to which the control groups are subjected to when using this model of IPF or PF given the aim of the study.

The ECM is a filamentous network composed of various fibres, and its mechanical response depends on the intrinsic mechanical features of said fibres and how they organise between themselves. When probing the mechanical response of the ECM using AFM, it is important to take into account the contact area between the probe and the sample, taking into consideration the dimensions of the fibres composing the network. This relationship determines if what is being measured is the stiffness of single fibres or the response of the interaction of entangled fibres in the network. With this in mind, we firstly carried out low-resolution elasticity measurements with AFM using a large spherical probe. This allowed us to obtain the largest contact area between the probe and the ECM. Taking into account the relationship between the diameter (D) of the contact area of a spherical tip and its indentation on a flat surface ($D=2{\left(r\delta \right)}^{1/2}$), at indentations between 0.6 and 1 μm using an 11 μm diameter tip, we achieve contact radii estimated at 3.6–4.7 μm. On the other hand, high-resolution maps using a probe with 75nm radius at indentations between 200 and 300 nm resulted in contact radii estimated at 173–212 nm. Thick collagen fibres show a thickness between 0.5 and 1.5 μm, suggesting that the higher values for E found via the high-resolution maps mainly account for the stiffness of the fibres, whereas the lower values for *E* at the low-resolution maps preferentially account for the stiffness resulting from the entanglement of the fibres, thus explaining the 1 order of magnitude difference in stiffness between both maps.

When looking into the differences in stiffness of EHR and ELR at day 7, we see that the differences between PBS- and bleomycin-treated samples follow the same relationship. This can be an indication that alterations during the initial inflammation phase happen at the single fibre level. At later time points of 14 and 28 days after instillation, we see that the values for EHR are kept high for the bleomycin-treated lungs but lower significantly for the PBS-treated lungs. On the other hand, the ELR maps follow the same tendency at the different timepoint with a switch at day 28. This suggests that, at the early stages of fibrosis, the main mechanical hallmark is the changes in stiffness of the individual fibres. Conversely, after day 14, a change in the supra-organisation of the fibres takes place at the network level, which is explained by the predominance of a stiffer fibrotic network at later stages. In fact, these results are in line with the morphological features observed through the QI images (Figure 6), where a thinning of the fibres is observed for the bleomycin-treated samples at day 14 and 28, whilst no noticeable difference is observed for the PBS-treated samples through time. The change in the evolution of the mechanical response of the network observed through the ELR maps likely arises from the changes in size and organisation of the fibres observed for the bleomycin-treated samples after day 14. Our results suggest that in IPF, this increase in stiffness is mainly observed at the fibre level in early stages and at the network level at later stages of fibrosis.

It is established that fibrosis, and in particular IPF, are characterised by an increase in collagen content in the ECM. However, several studies have reported that significant changes in total collagen content on the bleomycin-induced model of pulmonary fibrosis can only be seen at least 28 days after treatment [28,35]. A study on the mechanical properties of the ECM and tissue viscoelasticity in the bleomycin-induced model of pulmonary fibrosis reported that changes in mechanics were maximal before collagen content increased [28]. Recent studies have been looking into dissociating the increase in tissue stiffness to the simple increase in collagen content, and looking into the assembly and conformation of the fibres that constitute the ECM network [32,36]. It has been reported in fibrotic diseases an increase in content of fibronectin, and that higher concentrations of fibronectin delay the fibrillogenesis of type III collagen and accelerate that of type I [37]. In fact, in vivo collagen I assembly requires active fibronectin fibrillogenesis. Furthermore, studies showed that in established areas of scar formation, type I collagen is almost exclusively present, representing approximately 80% of the total collagen in IPF [38,39]. With this in mind, we looked into changes in the presence of fibronectin in ECM throughout the different time points. Interestingly, the evolution followed in relative fibronectin content through the different timepoints is analogous to the one observed in the ELR elasticity maps. Work reported by Kubow and colleagues [40] have shown that collagen I preferentially colocalizes with relaxed fibronectin fibres. Therefore, the mechanical behaviour of fibronectin fibrils likely influences the assembly or reorganisation of the ECM, translating into overall mechanical changes of the network. These results are in accordance with the results we found, suggesting that the relative amount of fibronectin directly influences network stiffness.

Elastin is the main fibre responsible for the elastic recoil of the lung, thus having a key role in determining the mechanical response of the ECM network, and, consequently, in the mechanosensing pathways of the populating cells. A simple model of the lung parenchyma uses a series of extensible springs represented by elastin fibres, and inextensible strings represented by collagen fibres [41]. As the lung is an organ which is constantly subjected to stretching due to breath work, elastin plays a key role in maintaining the homeostasis of the tissue. However, the role of elastin in the diseased lung, and particularly in fibrosis, still lacks understanding. Our results show an increase in elastin in the bleomycin-treated lungs with time, peaking at day 28, whilst in the PBS-treated lungs its content peaks at day 14 and is followed by a decrease. Thus, we can hypothesise that the presence of elastin might contribute to the turnover of the disease, as previous results reported by our lab suggested that there is a transition of behaviour from strain-hardening to strain-softening of the ECM that might work as a protection mechanism of the lung to prevent fibroblasts of proliferating after their initial activation [42]. The increase in elastin in the PBS-treated samples could be partly responsible for this turnover due to its physical role on the mechanical response of the lung ECM.

Micro-rheology allows us to study the mechanical behaviour of tissues beyond determining their stiffness. It allows for the characterization of the tissue in response to a load and determines its tendency to behave as a solid-like or liquid-like material. In fact, the lung parenchyma is among the most deformable tissues in the body, and resembles a foam material consisting of a solid phase and a fluid phase that closely interact with each other [43]. Previous reports from our laboratory on micro-rheology measurements of lung ECM have modelled the data using a double power-law [23]. However, due to instrumentation limitations in the range of frequencies that could be applied, the high-frequency exponent beta was fixed at 3/4 following previously reported work related to cytoskeleton dynamics [44]. Now, we are able to measure the response of decellularized lung ECM to a range up to $10^{4}$ Hz, allowing to leave also the second exponent as a free variable and assess its value experimentally.

The low-frequency exponent alpha is expected to be 0 for a purely elastic response, resulting in a plateau of G′, as predicted by semi-flexible filament theories. The very low values of the α exponent and β values higher than 0.5 for all days and conditions suggest the signature of an elastic plateau at low frequencies, followed by single filament relaxation at high frequencies, as predicted for semi-flexible filament networks and observed on reconstituted polymer networks [45,46,47]. The high-frequency exponent β is expected to be 1 for a purely viscous response, 0.75 for relaxed semi-flexible filament networks, and 0.5 for tensed semi-flexible filament networks [45,48]. As the lungs were measured at an inflation level corresponding to their functional residual capacity (FRC), the filaments in the network are expected to be pre-tensed to an extent corresponding to the FRC state. The exponent β fit close to 0.75 for the PBS-treated lungs at day 7, decreasing with time and coming closer to 0.5 at day 28. On the other hand, the bleomycin-treated lungs start at day 7 with a β close to 0.5 and increase through time. This response of the network is in agreement with the conformational changes on the network observed through the HR maps, which suggested that fibrosis leads to a disorganization and thinning of the fibres. Our previous reports on micro-rheology analysing the effects of stretch in the fibrotic ECM showed that stretch induces a fluidization of the matrix, suggesting a link to an inherent protection mechanism against fibrosis. These results are in accordance with the hypothesis, as we report a fluidization of the matrix throughout the development of fibrosis. Regarding the PBS-treated samples, a solidification of the ECM takes place with time after the resolution of the initial inflammatory state and mechanical homeostasis restoration. However, we note that there may be other important contributions which are not fully described by the double power-law, and may result in a more complex viscoelastic response, such as poroelasticity. In fact, studies have shown in other connective tissues, such as the tendon and the skin, that at low frequencies (<$10^{2}$ Hz) these assume a viscoelastic behaviour and at higher frequencies ($10^{2}$–$10^{4}$ Hz), there is a poroelastic behaviour instead [49,50]. These reports align with the architecture and porous nature of the lung. Physical forces are known to regulate cellular function. Particularly in tissue reparative response, physical forces and their corresponding cellular response have a determinant role in the processes that initiate and resolve injury. Fibrosis arises from a failure during the reparative response, which results in an aberrant deposition of ECM components and formation of scar tissue which alter the normal mechanosensing pathways experienced by the cells. These alterations result in the feeding of a positive feedback loop, which drives the progression of fibrosis. Recent studies have reported that substrate stiffening activates the fibroblast pro-fibrotic profile, contributing to the progression of fibrosis [14,51,52]. Furthermore, it has been reported that changes in the viscoelasticity of the ECM might be responsible for maintaining the activated state of cells that modulate the reparative response of the tissue [15]. The current most common and effective therapeutic agents used in slowing down the progression of IPF target the cellular processes that drive fibrosis. Amongst these are nintedanib and pirfenidone, that act on fibroblast activation and myofibroblast differentiation. However, they fail to reverse or stop the progression of IPF, showing to be efficient only in slowing down the process. Thus, there is still the need to look for more efficient targets that stop or reverse the progression of IPF. Targeting extracellular matrix mechanics has been shown to be a therapeutic approach with clinical potential [53]. Nakasaki and colleagues [54] have shown that the loss of Fibulin-5, an elastic fibre component, results in decrease in tissue stiffness and diminishes the inflammatory response, stopping the fibrotic phenotype progression in a mouse model of cutaneous fibrosis. These results are in line with our findings, suggesting that the components that act at the structural level of the ECM, influencing its assembly and organisation, present themselves as promising targets for the development of therapies to stop and resolve the progression of IPF. Overall, our findings suggest that the ECM goes through different stages throughout the development of IPF. These translate into different mechanical and organizational changes, which might be determinant in cell fate and disease progression, and can be used as targets in pharmacological studies and therapy design against IPF.

## 4. Materials and Methods

### 4.1. In Vivo Model of IPF

This experimental procedure was approved by the Ethical Committee for Animal Experimentation of the University of Barcelona and the Animal Experimentation Committee of regional authorities (Generalitat de Catalunya, OB 168/19 and 10972). Lungs were excised from N = 30 adult male Sprague Dawley rats (≈8 weeks old, 300g) (Charles River, MA, USA). IPF was induced by intratracheal instillation of bleomycin, as described elsewhere [55]. Animals were submitted to either intratracheal infusion of vehicle phosphate-buffered saline (PBS) (control group, *n* = 9) or bleomycin (fibrotic group, *n* = 21). Briefly, rats were anaesthetised (isofluorane inhalation), followed by the infusion of bleomycin (2U/kg; Sigma-Aldrich, St Louis, MO, USA) diluted in 75 μL of PBS into the tracheal lumen. Animals were cared for under normal living conditions until their sacrifice. Sacrifices were carried out at days 7 (*n* = 3 PBS, *n* = 7 Bleo), 14 (*n* = 3 PBS, *n* = 7 Bleo), and 28 (*n* = 3 PBS, *n* = 7 Bleo) after infusion. At the day of sacrifice, rats were anaesthetised with urethane and sacrificed by exsanguination. Lungs were extracted en bloc with the heart, pulmonary artery and trachea, infused with 3 mL of optimal cutting tissue medium (OCT compound, Sakura, CA, USA) in order to reach their functional residual capacity (FRC) [56] and stored at −80 ${}^{\circ }$C.

### 4.2. Decellularization and Sample Preparation

Lung samples were sectioned at ≈25 μm thickness using a cryostat (Leica CM 3050 S, Leica). Slices were placed onto a positively charged glass slide (Superfrost Plus; Thermo Fisher Scientific, Waltham, MA, USA) and stored at −20 ${}^{\circ }$C until use. The tissue slices were decellularized following a protocol previously described [57]. In brief, samples were left to thaw for 30 min at room temperature (RT) before undergoing sequential washes of decellularizing and washing solutions. They were firstly washed with PBS (Thermo Fisher Scientific, Waltham, MA, USA) for 20 min and with deionized water for 10 min to remove the OCT and to induce cell osmotic shock. Afterwards, the slices were submerged in solutions of 2% sodium deoxycholate (SDC) (Sigma-Aldrich, St Louis, MO, USA) and 0.03% DNAse (Sigma-Aldrich, St Louis, MO, USA) twice for 15 min and once time for 20 mins, respectively, intercalated by 3 washes of PBS of 5 mins. Finally, the samples were washed with PBS thrice in order to remove any remaining cellular debris, and kept submerged in PBS until further use for AFM measurements and immunohistochemical imaging.

### 4.3. AFM Measurements

AFM measurements were performed using a NanoWizard IV AFM (JPK, Bruker, Billerica, MA, USA) mounted on an inverted optical microscope Ti-Eclipse (Nikon, Tokyo, Japan). All measurements were made with the lung slice submerged in PBS at RT. Calibration of the spring constant and deflection sensitivity was completed for each cantilever and each experiment by using the SNAP approach [58]. Optical calibration was performed using JPK software direct camera overlay. Large airways and blood vessels were avoided for measurement.

To assess the overall macro-scale stiffness of the ECM, low-resolution (LR) force maps using a scan size of 8 × 8 pixel, 40 × 40 μm square, were acquired using SPC-200804-0,1 cantilevers with a silicon nitride 5.5um radius sphere attached to their end (Bruker, Billerica, MA, USA) and a pre-calibrated spring constant of 0.04 N/m. An initial force-indentation curve (F−δ) was acquired before each map to assess the deflection setpoint at which it was possible to keep an indentation depth between 600 and 1000 nm, using a ramp size of 5 μm and a ramping speed of 10 μm/s. Individual maps were acquired at 5 different locations per slide, at least 100 μm apart.

High-resolution (HR) QI maps were made using a scan size of 256 × 128 pixel, 40 × 40 μm square, using PFQNM-LC cantilevers with a paraboloid tip of 75 nm radius (Bruker, CA, USA) and a pre-calibrated spring constant of 0.13 N/m. An initial force curve was acquired before each map to assess the deflection setpoint at which it was possible to keep an indentation depth between 100 and 300 nm, using a ramp size of 1 μm and a ramping speed of 40 μm/s. Individual maps were acquired at 3 different locations per slide, at least 100 μm apart.

Micro-rheology measurements were performed using SAA-SPH-1UM cantilevers (Bruker, Billerica, MA, USA) with a resonant frequency of 11.39 kHz and pre-calibrated spring constant of 0.22 N/m. An individual measurement consisted in sequential oscillations applied at frequencies 60 Hz, 100 Hz, 600 Hz, 1000 Hz, 2000 Hz, 3050 Hz, and 6000 Hz, applied in random order, at an operating indentation of ≈500 nm with an oscillation amplitude of 50 nm. In total, 10 measurements at the same point at 5 different locations were made, at least 100 μm apart.

### 4.4. Data Processing

The Hertz’s contact model for a rigid sphere indenting a semi-infinite half-space was used to compute the Young’s modulus (*E*) from the obtained (F−δ) curves:

(1) $$F=\frac{4E}{3(1-\upsilon^{2})}R^{\frac{1}{2}}\delta^{\frac{3}{2}}$$

where δ is indentation, *R* is the radius of the sphere, and υ is the Poisson’s ratio, assumed to be 0.5. For each ramp, only the approach part of the force-indentation curves was fitted to Hertz’s model using JPK Data Analysis software, avoiding the substrate bottom effect by using indentations less than 10% of the sample thickness.

For micro-rheology measurements, the complex shear modulus ($G^{*}$) was determined from the force-indentation (F−δ) loops in the contact region by Fourier analysis. The total transfer function was computed was

(2) $$H_{tot}\left(f\right)=\frac{F(f)}{\delta (f)}e^{-i\phi }$$

where F(f) and δ(f) are the Fourier transforms of the force and deflection, respectively, and iϕ is the delay of the piezoactuator. We then computed the complex shear modulus from the Taylor expansion of the Hertz model of a rigid sphere indenting an elastic half space as:

(3) $$G^{*}\left(f\right)=H_{tot}\left(f\right)\frac{1-\upsilon }{4\sqrt{R\delta_{0}}}-2\pi ifb\left(0\right)$$

where $\delta_{0}$ is the operating indentation and *R* the tip radius. The 2πifb(0) term corresponds to the viscous drag hydrodynamic force correction [59]. $G^{*}$ can be represented in terms of its real part $G^{\prime }$, which corresponds to the storage modulus, and its imaginary part $G^{\prime \prime }$, which corresponds to the loss modulus. The storage modulus accounts for the stored elastic energy, whilst the loss modulus accounts for the dissipated energy. The loss tangent represents the relationship between these terms and is computed as:

(4) $$\eta =\frac{G^{{}^{\prime \prime }}}{G^{{}^{\prime }}}$$

which is an index of the solid-like (<1) or liquid-like (>1) behaviour of the sample. $G^{*}\left(f\right)$ was then modelled to a linear superposition of two power-laws as:

(5) $$G^{*}\left(f\right)=A{\left(if\right)}^{\alpha }+B{\left(if\right)}^{\beta }$$

from which the first term assumes a low-frequency regime characterised by a weak power-law, and the second term accounts for the high-frequency regime. The algorithms to obtain G* from micro-rheological measurements were implemented in Python 3 using the resources provided by the SciPy library [60] for signal analysis. Model fits were performed using the open-access user-friendly online platform fitteia [61] (https://fitteia.org, accessed on 22 November 2022) that uses the non-linear least-squares minimization method with a global minimum target, provided by the numerical routine MINUIT from the CERN library [62].

**Figure 10:**
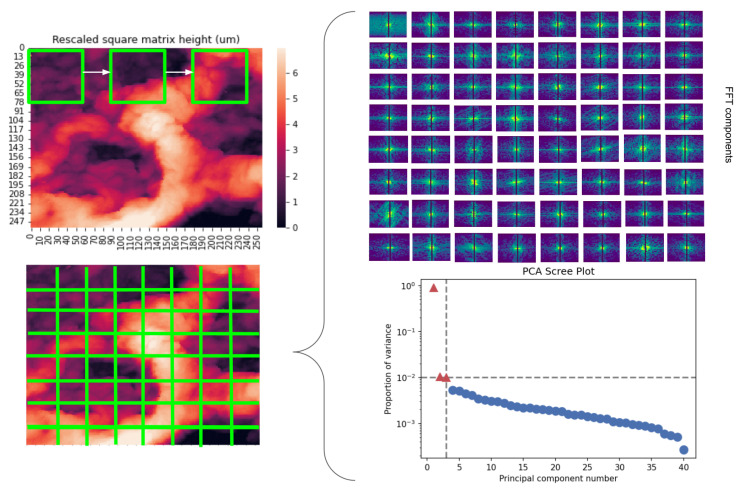
Graphical presentation of the height image RPCA analysis method. The height image is split in tiles (**left**) from which each FFT spectrum is computed (**top right**). The spectrums are fed to the RPCA algorithm from which we can obtain the number of components from the PCA scree plot (**bottom right**).

To analyse the QI maps, the height and its corresponding elasticity image were considered. Initially, all images were resized to have a squared dimension of 256×256 pixel. For the elasticity images, each pixel corresponds to a value of *E* of the computed (F−δ) curve. Regarding height images, each pixel corresponds to the cantilever position (*z*) at the identified tip-sample contact point. With the goal to correlate specific local topographical features with its corresponding *E*, a tiling of the images was made with a window size of 32×32 pixel. This results in an image divided into 8×8 tiles. Geometric mean and median values per tile were calculated from the elasticity maps. The surface roughness of the ECM was determined by calculating the arithmetic average of profile height deviations from the quadratic average of profile height deviations from the mean line ($R_{q}$) of the height image [63]. Moreover, to assess which features of the height images were inducing the most variability to the overall morphological profile of the measured area, the Fourier spectrum of each tile of the height map was calculated. These were then fed to a robust principal component analysis [64] algorithm, from which a certain number of components were identified in each image (Figure 10). This approach was based on the method developed by Jany and colleagues [65] for the automatic analysis of microscopic images. Image processing and analysis was performed using custom programs written in Python 3 resourcing to the implementations from Scikit-Learn library as in Hyperspy toolbox [66].

#### 4.4.1. Immunohistochemical Imaging

Fluorescent immunostaining of decellularized lung slices was carried out to determine the structure and composition of the ECM. Samples were fixed using paraformaldehyde (PFA) 4% for 15 min at RT and washed 3 times for 5 mins with PBS. Samples were blocked using a blocking solution composed of 10% fetal bovine serum (FBS) and 3% bovine serum albumin (BSA) for 1 h at RT at a low orbital agitation (80 rpm). Samples were incubated overnight (≈16 h) at a constant orbital agitation (80 rpm) in 4 ${}^{\circ }$C with primary antibodies against elastin (1:100, mouse anti-elastin, sc-58756, Santa Cruz Biotechnology) and fibronectin (1:100, rabbit anti-fibronectin, ab2413, Abcam). After removing the primary antibodies with 3 washes of 10 min each of the blocking solution, secondary antibodies (1:200, Goat Anti-Rabbit Cy3, ab97075, Abcam) and (1:200, Goat Anti-Mouse Alexa fluor 488, AB_2536161, Thermo Fisher Scientific, Waltham, MA, USA) were added and left for 2 h at 37 ${}^{\circ }$C and at 80 rpm. Finally, nuclear counterstain Hoechst 33342 (NucBlue™Live ReadyProbes™Reagent, Thermo Fisher, Invitrogen) was added and left for 20 min at RT, followed by three 5-min washes with PBS. The slides were mounted using fluoromount mounting media (Thermo 295 Fisher Scientific, Waltham, MA, USA). Epifluorescence images were acquired with a Leica SP5 inverted microscope equipped with a cooled CCD camera (C9100, Hamamatsu Photonics K.K., Hamamatsu, Japan) using a 20X Plan Fluor objective (Nikon, Tokyo, Japan). Exposure time was kept constant for all the images throughout the acquisition and images were analysed using custom-developed scripts [67].

#### 4.4.2. Statistical Analysis

For the ELR and EHR maps, data displayed a lognormal distribution, so we carried out two-tailed paired *t*-tests to the logarithm of the data. Each mapped region was considered as independent and was taken as N for statistical analysis. For the remaining data, which displayed a normal distribution, two-tailed paired *t*-tests were performed on the original data and N was considered as number of animals. Statistical tests were performed using Python 3 resourcing to the implementations from SciPy library [60].

## Figures and Tables

**Table 1:** Parameters of a two-power law and estimated transition frequency. The parameters were fitted to data acquired for the PBS and bleomycin-instilled lungs at 7, 14, and 28 days after instillation. The transition frequency $f_{t}$ was defined as the frequency at which $A{\left(if\right)}^{\alpha }=B{\left(if\right)}^{\beta }$, that is, $f_{t}=exp\left(\frac{ln(B)-ln(A)}{\alpha --\beta }\right)$. Values are shown as mean ± SD.

		A (Pa)	α	B (Pa)	β	$f_{t}$ (Hz)
	7	3210±793	$10^{-13}\pm 0.03$	52±21	0.72±0.05	309
PBS	14	3533±1214	0.02±0.15	70±81	0.67±0.13	404
	28	4516±1361	$10^{-11}\pm 0.10$	181±111	0.55±0.07	356
	7	8579±3142	$10^{-07}\pm 0.69$	193±212	0.53±0.14	1221
BLEO	14	4671±1216	$10^{-12}\pm 0.06$	85±48	0.64±0.08	522
	28	5087±1341	$10^{-11}\pm 0.19$	79±49	0.65±0.08	583

## Data Availability

Data available upon request.
